# Dietary creatine intake and depression risk among U.S. adults

**DOI:** 10.1038/s41398-020-0741-x

**Published:** 2020-02-03

**Authors:** Amanda V. Bakian, Rebekah S. Huber, Lindsay Scholl, Perry F. Renshaw, Douglas Kondo

**Affiliations:** 1 Department of Psychiatry, University of Utah School of Medicine, Salt Lake City, UT USA; 2 The Rocky Mountain Veterans Integrated Service Network 19 Mental Illness Research, Education, and Clinical Centers of Excellence, Salt Lake City, UT USA

**Keywords:** Human behaviour, Depression

## Abstract

Creatine monohydrate is actively being researched for its antidepressant effects, yet little is known about the link between dietary creatine and depression risk. This study examines the association between dietary creatine and depression in U.S. adults, using data from the 2005 to 2012 National Health and Nutrition Examination Survey (NHANES). Patient health questionnaire, dietary creatine intake and covariates were obtained on 22,692 NHANES participants ≥20 years of age. Depression prevalence was calculated within quartiles of dietary creatine intake. Adjusted logistic regression models were formulated to determine the relationship between dietary creatine intake and depression risk. Additional covariates included income to poverty ratio, race/ethnicity, sex, age, education level, body mass index, healthcare access, smoking status, physical activity, and antidepressant/anxiolytic medication use. Models were further stratified by sex, age group, and antidepressant/anxiolytic medication use. Depression prevalence was 10.23/100 persons (95% CI: 8.64–11.83) among NHANES participants in the lowest quartile of dietary creatine intake compared with 5.98/100 persons (95% CI: 4.97–6.98) among participants in the highest quartile (*p* < 0.001). An inverse association was measured between dietary creatine and depression (adjusted odds ratio (AOR) = 0.68, 95% CI: 0.52–0.88). Dietary creatine’s negative association with depression was strongest in females (AOR = 0.62, 95% CI: 0.40–0.98), participants aged 20–39 years (AOR = 0.52, 95% CI: 0.34–0.79) and participants not taking antidepressant/anxiolytic medication (AOR = 0.58, 95% CI: 0.43–0.77). Study results indicate a significant negative relationship between dietary creatine and depression in a nationally representative adult cohort. Further research is warranted to investigate the role creatine plays in depression, particularly among women and across the lifespan.

## Introduction

Major depressive disorder (MDD) affects 16.2% of all Americans at some point during their lifetime^1^ and is the leading contributor to disability worldwide^2^. MDD’s escalating public health relevance and the modest efficacy of currently available treatments have prompted efforts to identify modifiable patient factors associated with depression onset, chronicity, and severity. Substance use^3^, childhood adversity^4^, unemployment^5^, physical inactivity^6^, medical comorbidity^7^, insufficient social support^8^, and poor nutrition^9^ are among the potentially modifiable risk factors most consistently linked with MDD.

Recent population-based studies report protective effects of a healthy diet against depression^10^ suggesting that dietary change or nutritional supplementation may provide a viable prevention or intervention strategy for MDD. In particular, public health initiatives aimed at increasing consumption of specific nutrients such as iodine fortified salt and folic acid supplemented grain products demonstrate the positive impact that a population-level, diet-based intervention may have on disease prevention^11^. While nutritional medicine has gained substantial attention in psychiatry^12^, specific nutrients’ influences on depression risk have yet to be elucidated fully. Several nutrients with known neurobiological activity that may influence mood are being investigated as monotherapeutic and adjunctive therapies for MDD. A recent meta-analysis of clinical antidepressant trials support the therapeutic use of S-adenosylmethionine, methylfolate, omega-3 fatty-acids (primarily eicosapentaenoic acid (EPA) or ethyl-EPA), and vitamin D. Results were mixed for zinc, folic acid, vitamin C, and tryptophan, and negative for inositol^13^.

Creatine, a nitrogenous organic acid endogenous to all vertebrates, plays a critical role in brain bioenergetics^14,15^ and is another promising nutraceutical candidate for depression. In humans, creatine is continuously replenished through de novo synthesis and diet. A small proportion of creatine’s homeostatic load (1.7%, or about 2 g, for an average adult) is excreted daily in the urine as creatinine. Replenishing this lost creatine through diet would require an average individual to consume ~500 g of raw meat per day^14^.

Early evidence of creatine’s potential efficacy as an antidepressant emerged during a pilot randomized clinical trial for Parkinson disease (PD). Compared with placebo, creatine was associated with a significant reduction in depressive symptoms in PD patients following a 2-year treatment course^16^. Recently, creatine monohydrate has been studied as an adjunctive treatment for depression with antidepressants in open-label^17–20^ and randomized clinical depression trials^21–23^. These studies have largely^17–22^, but not entirely^23^, demonstrated that creatine supplementation enhances and/or accelerates antidepressant response. Of note, the majority of clinical creatine studies have focused on antidepressant augmentation in adolescent and adult women. This sex-specific pharmacologic treatment strategy is supported by creatine’s stronger antidepressant-like effects in female rodent models^24,25^ as well as magnetic resonance spectroscopy research suggesting that the link between abnormal brain energy metabolism and depression is more common in women than men^26^.

Epidemiologic studies measuring the association between the dietary consumption of specific nutrients and depressive symptoms provide critical insight into possible prevention efforts for depression while justifying more finely-focused animal studies and human intervention trials. While observational research has examined the relationship between specific nutrients including omega-3 fatty acids^27,28^ and dietary B vitamins^29–31^ and depression, the association between dietary creatine intake and depression risk is currently unknown. Based on reports of creatine’s antidepressant effects independent^16^ of and in combination^17–22^ with antidepressant administration, the current study extends this line of investigation to a large, population-based, U.S. sample to determine creatine’s relationship with depression within the context of the North American diet. Using data from the Centers for Disease and Control and Prevention’s National Health and Nutrition Examination Survey (NHANES), we (1) measured the prevalence of depression among community-dwelling adults in the U.S. across quartiles of dietary creatine intake, (2) examined the relationship between average daily dietary creatine intake and self-reported symptoms of depression, and (3) investigated potential modification of the association between average daily dietary creatine intake and depression by sex, age, and antidepressant/anxiolytic medication use.

## Materials and methods

### Study population

Data for this study was acquired from the continuous NHANES 2005–2012 for adult participants 20–85+ years of age. NHANES is administered through the Centers for Disease Control and Prevention’s National Center for Health Statistics (NCHS) and is designed to collect health and nutritional measurements on an annual basis from a nationally representative civilian, noninstitutionalized U.S. sample. The survey includes self-reported questionnaires and a physical examination (e.g. medical, laboratory, and dental measurements) collected at a mobile exam center [MEC]). As the specific health and nutritional measurements collected by NHANES are subject to change from year to year, 2005–2012 was selected to ensure a high consistency of variables across individual surveys as well as sufficient study power. Approval to conduct NHANES was granted by the NCHS Research Ethics Review Board through protocols #2005–2006 and #2011–2017 and informed consent was obtained from all participants.

### Depression

The NHANES depression screening questionnaire consists of nine items derived from the Patient Health Questionnaire (PHQ-9); all MEC participants were eligible to receive the depression screener. The PHQ-9 is a validated and reliable^32^ self-report nine-item assessment tool that coincides with criteria for a major depressive episode according to the American Psychiatric Association’s Diagnostic and Statistical Manual (DSM) of Mental Disorders 4th edition. These criteria have not changed substantively in subsequent DSM editions^33^ and remain applicable for the NHANES cohort. Total PHQ-9 scores range from 0 to 27 with the following threshold scores established for depression severity: mild (5), moderate (10), moderately severe (15), and severe (20). A PHQ-9 score of ≥10 has yielded 88% sensitivity and 88% specificity^34^ for identifying MDD and was therefore selected as the binary threshold to define the presence of depression in this cohort.

### Dietary creatine

Animal-based proteins (i.e., meat, poultry, fish, and seafood) are the largest contributors of creatine to the human diet. Individual-level information on dietary intake of animal protein was obtained from NHANES’s dietary interview first and second day questionnaires, which aim to record a participant’s total dietary intake during two discrete 24-hour periods. The first day dietary interview was conducted in-person during the MEC assessment and the second day dietary interview was performed 3–10 days later over the phone. The ounces of creatine-rich meat and fish recorded during the dietary interview and labeled using U.S. Department of Agriculture food codes were organized into the following MyPyramid Equivalents^35^ database subgroups: (1) meat (beef, pork, veal, lamb, and game), (2) organ meats (from meat and poultry), (3) frankfurters, sausage, and luncheon meats, (4) poultry (chicken, turkey, and other), (5) fish and shellfish high in n-3 fatty acids, and (6) fish and shellfish low in n-3 fatty acids. The grams of creatine/ounce of the individual meat items included in the MyPyramid subgroups were estimated based on extensive literature review^36–45^ (also see Supplementary Materials). Creatine concentrations ranged from 0.06 g/oz in frankfurters, sausage, and luncheon meats to 0.16 g/oz in fish high in n-3 fatty acids (Supplementary Tables S1 and S2). The estimated concentration of creatine averaged across all sources of animal-based protein (i.e. beef, pork, veal, lamb, game, organ meats, frankfurters, sausages, luncheon meat, poultry, fish, and shellfish) was 0.11 g/oz. The average grams of creatine consumed across the 2-day dietary interview was calculated and used as the primary exposure in the analyses. An additional exposure examined 2-day average dietary creatine intake (“dietary creatine”) categorized into quartiles.

### Covariates

Potentially confounding and/or moderating demographic, socioeconomic, and lifestyle factors were identified based on prior research^1^ and clinical expertize, and were downloaded from the NHANES 2005–2012 database including family income to poverty ratio (i.e. ratio of family income to the family’s poverty threshold according to the U.S. Census Bureau based on family size and composition), race/ethnicity, sex, maximum achieved education level, age, body mass index (BMI), smoking status (current, previous, or never), whether or not the participant has a routine place to go for healthcare, total moderate-to-vigorous minutes of physical activity per week, and antidepressant/anxiolytic medication use.

### Statistical analyses

NHANES’ complex sampling design was incorporated into all analyses. Missing data patterns were investigated using the %missingPattern macro^46^ in SAS and missing data were determined to meet missing at random assumptions. Multiple imputation was conducted for missing numeric and categorical variables to minimize bias and maximize precision and power. Ten imputations of missing data were generated using the chained equation approach^47^. Depression prevalence was calculated across the sample and within quartiles of dietary’ creatine intake. *T*-tests and Rao-Scott adjusted chi-square tests were used to compare characteristics between persons with and without depression. Single (crude) and multivariable logistic regression models were fit to determine the relationship between dietary creatine intake and depression risk. Multivariable logistic regression models were adjusted for potential confounding including income to poverty ratio, race/ethnicity, sex, age, education level, BMI, healthcare access, smoking status, total moderate-to-vigorous minutes of physical activity per week, and psychotropic medication use. Models were stratified by sex, age group (20–39 years, 40–64 years, ≥65 years) and antidepressant/anxiolytic medication use to examine effect modification. Additional models were fit for the entire population and stratified by sex in which creatine exposure was categorized into quartiles using the lowest quartile as the reference category. Statistical analyses were conducted in SAS version 9.4 (SAS Institute Inc., Cary, NC), an alpha of 0.05 was used to assess statistical significance, and all statistical tests were two-sided.

### Sensitivity analysis

Dietary creatine intake is correlated with the consumption of additional nutrients found in animal-based protein that are also associated with reducing depression risk including n-3 fatty acids. Oily fish is the primary dietary source of long chain n-3 fatty acids^48^. The average ounces of fish high in n-3 fatty acids and the average ounces of total fish consumed across 2 days were calculated for each participant based on responses to the dietary interview first and second day questionnaires. To determine how robust study findings were to adjustment with dietary fish sources of n-3 fatty acids, additional adjusted logistic regression models were formulated that included (1) the average ounces each participant consumed of fish high in n-3 fatty acids and (2) the average ounces each participant consumed of total fish.

Supplemental creatine, typically in the form of creatine monohydrate, is an additional source of exogenous creatine that is primarily consumed to improve athletic performance and build muscle strength. The 1-week prevalence of supplemental creatine use in the adult U.S. population is estimated to be 0.9%^49^. Participants taking supplemental creatine within the 30 days preceding the NHANES survey were identified using the Dietary Supplement Questionnaire. A sensitivity analysis was performed in which participants supplementing their dietary creatine intake were excluded from the analysis.

## Results

### Depression prevalence and creatine consumption

**Fig. 1:**
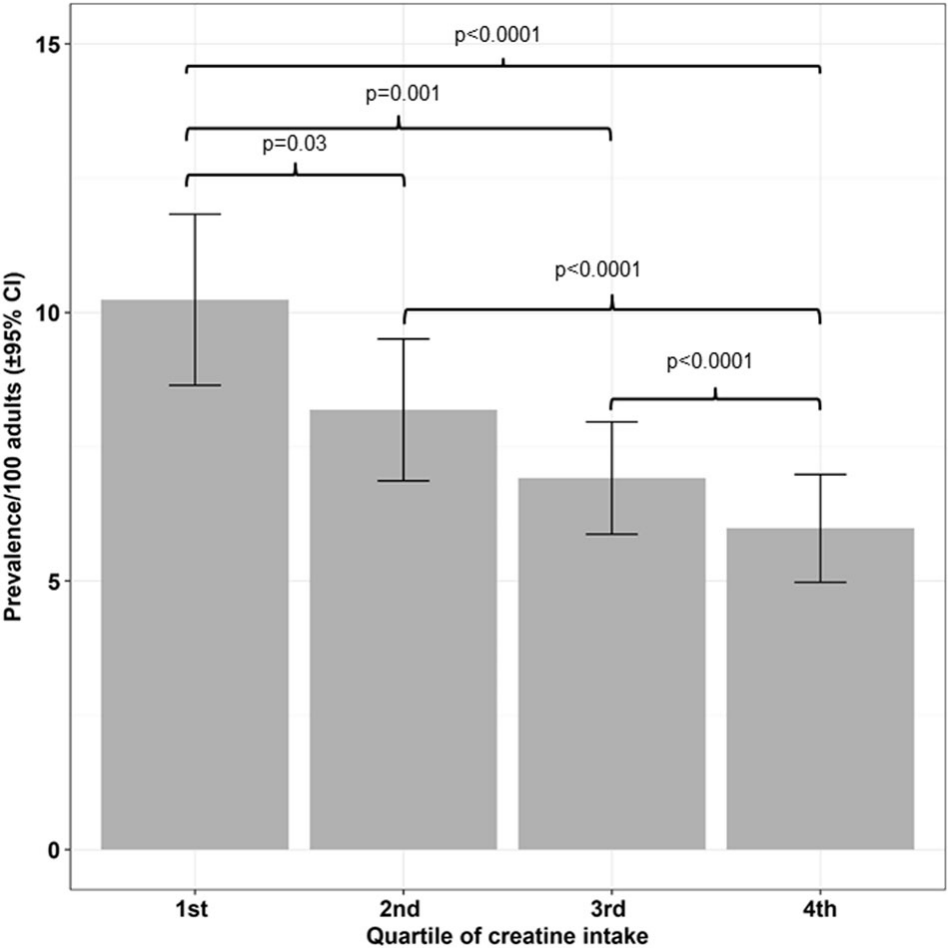
Crude prevalence (weighted percentages are presented) and 95% confidence interval of depression (defined as a PHQ-9 score of ≥10) by quartile of 2-day average dietary creatine intake among NHANES 2005–2012 adult participants.

The NHANES 2005–2012 sample included 22,692 adult participants 20 years of age or older. Depression screener data was available for 19,361 adult participants (*N*_males_ = 9549; *N*_females_ = 9812); 1696 participants screened positive for MDD (Table 1). The overall weighted prevalence of depression among NHANES 2005–2012 adult participants was 7.33/100 persons (95% confidence interval (CI): 6.65–8.00). Two-day average dietary creatine intake was available for 18,092 participants. Depression screener data were available for 16,816 adult participants with dietary data; 1455 of these participants screened positive for depression. The weighted prevalence of depression among participants with dietary data was 7.71/100 persons (95% CI: 6.91–8.50). Average creatine intake across all participants was 0.54 g (standard deviation (SD) = 0.35 g). Average creatine intakes for males and females were 0.67 g (SD = 0.39 g), and 0.42 g (SD = 0.26 g), respectively. Two-day average dietary creatine intake was categorized into quartiles ranging from 0–0.26 g (1st quartile; mean = 0.15 g, SD = 0.08 g), 0.27–0.45 g (2nd quartile; mean = 0.36 g, SD = 0.05 g), 0.46–0.69 g (3rd quartile; mean = 0.57 g, SD = 0.07 g), and 0.70–3.16 g (4th quartile; mean = 1.01 g, SD = 0.30 g). Mean dietary creatine intake differed significantly between participants with and without depression (0.48 g; SD = 0.33 g versus 0.54 g; SD = 0.35 g, respectively, *p* < 0.0001; Table 1). Figure 1 illustrates the stepwise, inverse relationship between depression prevalence and quartile of dietary creatine consumption. Depression prevalence was 10.23/100 persons (95% CI: 8.64–11.83) among participants in the lowest quartile of dietary creatine consumption compared to 5.98/100 persons (95% CI: 4.97–6.98) among participants in the highest quartile of dietary creatine consumption (*p* < 0.0001).

**Table 1:** Characteristics of adult NHANES 2005–2012 participants with PHQ-9 data (*N* = 19,361) (Table does not include imputed data).

	Depressed (*N* = 1696)		Not Depressed (*N* = 17,665)		
Characteristic	Weighted (%)	95% CI	Weighted (%)	95% CI	*P* value
Education^a^					<0.0001
<9th grade	9.66	7.94–11.38	5.44	4.78–6.10	
9–11th grade	19.7	17.27–22.14	11.28	10.08–12.48	
High school diploma/GED	27.18	24.25–30.12	23.08	21.85–24.30	
Some College/AA degree	31.98	28.87–35.08	31.00	29.81–32.20	
≥College graduate	11.48	8.45–14.51	29.21	27.04–31.37	
Sex^a^					
Female	64.07	61.58–66.55	50.21	49.47–50.96	<0.0001
Male	35.93	33.45–38.42	49.79	49.04–50.53	
Age group					
20–39 years	34.77	32.20–37.34	36.92	35.08–38.75	<0.0001
40–64 years	54.90	52.36–57.44	45.26	43.84–46.67	
≥65 years	10.33	8.79–11.87	17.83	16.67–18.99	
Race/ethnicity^a^					<0.0001
Mexican American	8.37	5.82–10.91	7.96	6.36–9.56	
Other Hispanic	7.95	5.15–10.74	4.61	3.52–5.71	
Non-Hispanic White	63.36	57.85–68.87	70.72	67.42–74.01	
Non-Hispanic Black	15.07	12.02–18.13	10.67	8.93–12.42	
Other	5.26	3.92–6.60	6.04	5.16–6.91	
Healthcare place^a^	86.86	84.51–89.20	85.76	84.85–86.67	0.32
Antidepressant or anxiolytic use^a^	36.81	33.15–40.47	11.06	10.34–11.77	<0.0001
Quartile creatine intake^a^					<0.0001
1st	27.88	23.92–31.84	20.42	19.25–21.59	
2nd	27.86	24.37–31.35	26.09	25.16–27.02	
3rd	24.45	21.33–27.56	27.47	26.44–28.51	
4th	19.81	17.00–22.62	26.01	24.89–27.14	
Smoking status^a^					<0.0001
Current smoker	41.14	37.79–44.48	20.14	19.04–21.25	
Former smoker	20.4	17.34–23.46	25.23	24.03–26.43	
Never smoker	38.46	34.63–42.30	54.63	53.15–56.11	
	Mean	SD	Mean	SD	
BMI^b^	30.30	7.97	28.62	6.56	<0.0001
Family income to poverty ratio^b^	2.06	1.56	3.11	1.62	<0.0001
Average creatine intake^b^, g	0.48	0.33	0.54	0.35	<0.0001
Total moderate-to-vigorous minutes of physical activity per week	478.37	851.98	627.46	952.91	<0.0001

*BMI* body mass index, *CI* confidence interval, *SD* standard deviation.

^a^Rao-Scott adjusted chi-square test.

^b^*t*-test.

### Participant characteristics by depression status

Comparisons of characteristics between participants with and without depression are shown in Table 1. Participants with depression were more likely to be female (64 vs. 50%; *p* < 0.0001), have a higher BMI (mean = 30.30 vs. 28.62, *p* < 0.0001), participate in fewer minutes of moderate-to-vigorous physical activity per week (mean = 478.37 vs. 627.46, *p* < 0.0001), currently smoking (41 vs. 20%, *p* < 0.0001), use antidepressant or anxiolytic medication (37 vs. 11%, *p* < 0.0001), and have a lower family income to poverty ratio (mean = 2.06 vs. 3.11, *p* < 0.0001). Statistically significant differences between participants with and without depression were also found in the weighted percent of race/ethnicity categories (*p* < 0.0001), age groups (*p* < 0.0001), and quartile measures of 2-day average dietary creatine intake (*p* < 0.0001). Having access to a routine place to receive healthcare was the only characteristic examined that did not differ significantly between participants with and without depression (87 vs. 86%, respectively; *p* = 0.32).

### Association of creatine with depression

Table 2 reports the odds of depression associated with 2-day dietary creatine intake among NHANES participants and stratified by participant characteristics including sex, age group, and antidepressant/anxiolytic use. Two-day average dietary creatine was highly protective against depression in the crude model (odds ratio (OR) = 0.54, 95% CI: 0.42–0.69, *p* < 0.0001). The relationship between odds of depression and dietary creatine intake across the entire sample was robust to model adjustment by likely confounders including age, sex, education, race/ethnicity, access to a routine place to receive healthcare, BMI, antidepressant or anxiolytic medication use, family income to poverty ratio, total moderate-to-vigorous minutes of physical activity per week, and smoking status (adjusted OR = 0.68, 95% CI: 0.52–0.88, *p* = 0.004). The adjusted models stratified by sex demonstrated a protective association between dietary creatine intake and depression risk among females (adjusted OR = 0.62, 95% CI: 0.40–0.98, *p* = 0.04) and although the adjusted odds ratio is below one, the relationship failed to reach statistical significance among males (adjusted OR = 0.72, 95% CI: 0.49–1.05, *p* = 0.08). Similarly, the age-group stratified models demonstrated evidence of modification with a significant association measured among participants 20–39 years of age (adjusted OR = 0.52, 95% CI: 0. 0.34–0.79, *p* = 0.002) but not among participants 40–64 (adjusted OR = 0.85, 95% CI: 0.57–1.25, *p* = 0.40) or ≥ 65 (0.66, 95% CI: 0.35–1.23, *p* = 0.19) years of age. A significant inverse relationship was identified between dietary creatine and depression risk among individuals not taking antidepressant or anxiolytic medication (adjusted OR = 0.58, 95% CI: 0.43–0.77, *p* < 0.0001). The relationship between dietary creatine and depression risk was not statistically significant among participants taking antidepressant or anxiolytic medication (adjusted OR = 0.78, 95% CI: 0.47–1.30, *p* = 0.35).

**Table 2:** Association between 2-day average dietary creatine intake and risk of depression among adult NHANES 2005–2012 participants (models include imputed data).

	Crude model		Adjusted model^a^	
Population	Odds ratio (95% CI)	*P* value	Odds ratio (95% CI)	*P* value
Entire population	0.54 (0.42–0.69)	<0.0001	0.68 (0.52–0.88)	0.004
Sex stratified models				
Males^b^	0.63 (0.45–0.90)	0.01	0.72 (0.49–1.05)	0.08
Females^b^	0.65 (0.40–1.03)	0.07	0.62 (0.40–0.98)	0.04
Age-group stratified models				
20–39 years^c^	0.38 (0.24–0.60)	<0.0001	0.52 (0.34–0.79)	0.002
40–64 years^c^	0.61 (0.43–0.86)	0.005	0.85 (0.57–1.25)	0.40
≥65 years^c^	0.34 (0.16–0.71)	0.004	0.66 (0.35–1.23)	0.19
Antidepressant/anxiolytic use models				
Yes^d^	0.74 (0.46–1.19)	0.22	0.78 (0.47–1.30)	0.35
No^d^	0.63 (0.48–0.83)	0.001	0.58 (0.43–0.77)	0.0002

*CI* confidence interval.

^a^Models adjusted for income to poverty ratio, race/ethnicity, sex, age, education level, BMI, healthcare access, smoking status, total moderate-to-vigorous minutes of physical activity per week, and antidepressant/anxiolytic medication use.

^b^Adjusted models do not include sex.

^c^Adjusted models do not include age.

^d^Adjusted models do not include antidepressant/anxiolytic use.

**Fig. 2:**
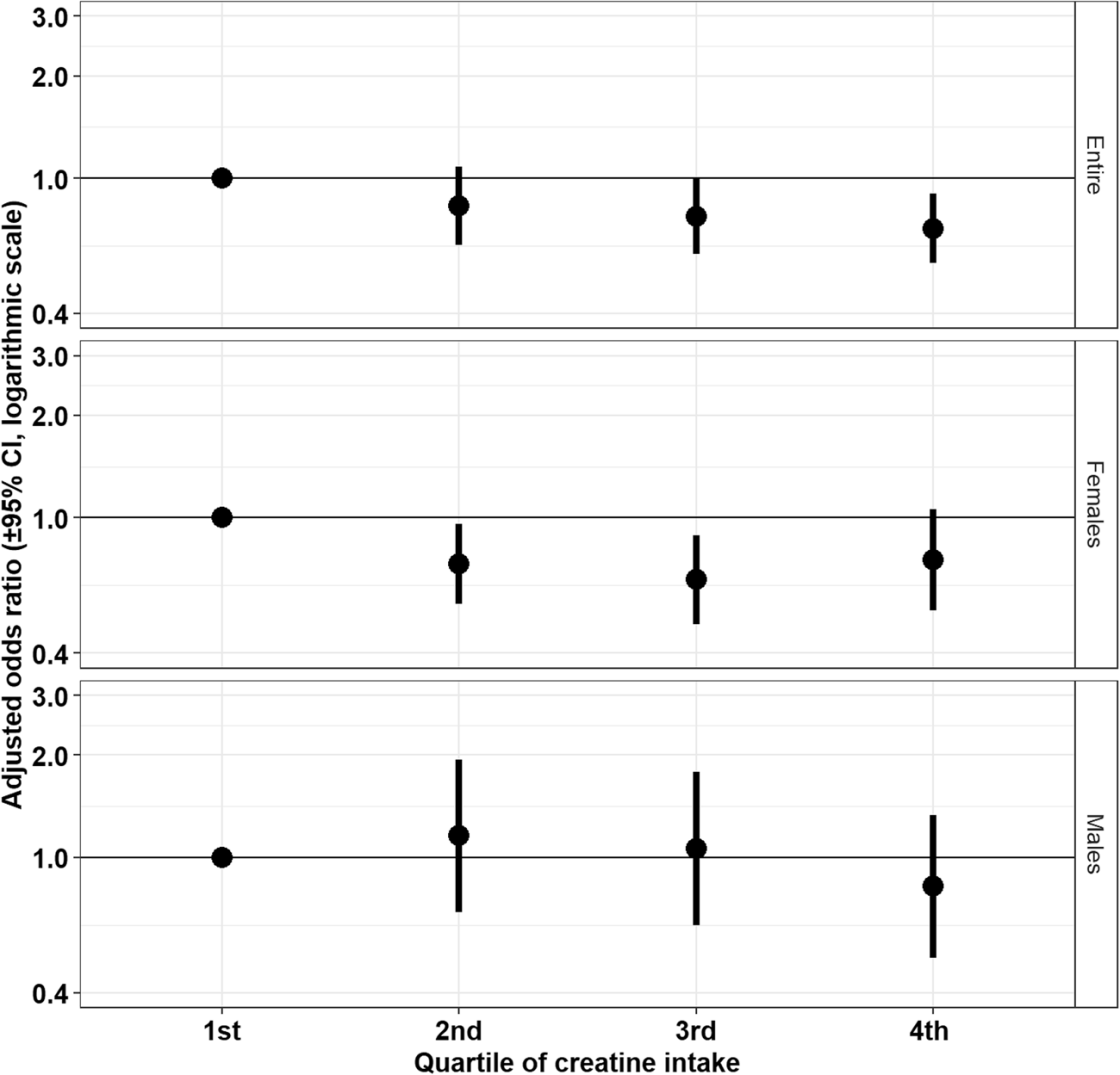
**Relationship (adjusted odds ratio and 95% confidence interval) between quartile of dietary creatine intake and depression risk among NHANES 2005–2012 participants stratified by sex.**

The association between quartiles of 2-day dietary creatine consumption and risk of depression are displayed in Fig. 2. Across the study population, dietary creatine was protective against depression among participants in the third (adjusted OR = 0.77, 95% CI: 0.60–0.99, *p* = 0.04) and fourth (adjusted OR = 0.71, 95% CI: 0.56–0.90, *p* = 0.005) quartiles of creatine consumption relative to the first quartile. Similarly, in the multivariable models stratified by sex, dietary creatine was found to protect against depression among females exposed to the second (adjusted OR = 0.73, 95% CI: 0.56–0.96, *p* = 0.02), and third (adjusted OR = 0.66, 95% CI: 0.49–0.89, *p* = 0.003) quartiles of dietary creatine relative to the first quartile while no statistically significant associations were measured in males regardless of creatine quartile.

### Sensitivity analyses

The relationship between dietary creatine and depression in the study population was robust to additional adjustment by participant’s consumption of 2-day average ounces of fish high in n-3 fatty acids (OR = 0.70, 95% CI: 0.54–0.92, *p* = 0.01) and 2-day average ounces of total fish (OR = 0.70, 95% CI: 0.52–0.94, *p* = 0.02; Supplementary Table S3).

The weighted prevalence of supplemental creatine use among participants was 0.09% (95% CI: 0.04–0.15%). No substantive differences emerged when participants taking supplemental creatine (*N* = 12) were removed from the adjusted logistic regression model (results not shown).

## Discussion

To our knowledge, this is the first study to examine the relationship between dietary creatine intake and risk of depression in a nationally representative U.S. sample. We found that MDD prevalence among U.S. adults follows a step-wise decrease corresponding to an incremental increase in dietary creatine consumption. Depression prevalence was 42% higher among adults in the lowest quartile (0–0.26 g) compared to adults in the highest quartile (0.70–3.16 g) of creatine consumption. In comparison, depression prevalence among persons with low creatine intake mirrors what has been estimated among persons with chronic medical conditions such as Type 2 Diabetes Mellitus^50^. After controlling for demographic and lifestyle variables, the risk of screening positive for depression was 31% lower among adults in the highest, compared to the lowest, quartile of creatine consumption.

Creatine, a nitrogenous organic acid that is synthesized naturally in the body and also acquired through diet, may mitigate pathophysiologic components of depression through its role as a brain energy buffer, anti-oxidant, and neuroprotectant. Adults with MDD demonstrate alterations in a brain bioenergetics system for which creatine, in the form of creatine phosphate, is a substrate^51^, facilitating rapid regeneration of adenosine triphosphate (ATP) in tissues with variable energy demands, such as muscle and brain^14^. Given creatine’s essential role in brain energy homeostasis, creatine supplementation is actively being explored for the treatment of depression in combination with antidepressant therapy with mechanistic support and efficacy demonstrated in both animal^24,25,52–57^ and human^16–22^ studies. Pooled estimates of creatine’s efficacy as an antidepressant across multiple human clinical trials have yet to be calculated.

The current study found evidence of a sex dimorphism in the relationship between dietary creatine intake and depression. Although 2-day dietary creatine was strongly associated with reduced depression risk across the entire study population, the statistical significance and strength of this association differed between men and women. The inverse, stepwise relationship between creatine intake and depression risk shown in Fig. 2 appears to be driven predominantly by female participants. The odds of depression were 18% lower for each increasing gram of creatine consumed by women compared to the total population. Current study findings support research from animal studies that indicate sex-specific variation in creatine’s antidepressant efficacy. Among Sprague-Dawley rats treated with supplemental creatine, female rats exhibited greater reduction in depressive and anxious behaviors than male rats^24,25^. Furthermore, human intervention studies demonstrate creatine’s efficacy in reducing depression symptoms in women when augmenting antidepressants. Eight weeks of supplemental creatine use with antidepressant therapy reduced depressive symptoms among female adolescents^17,19,21^ and adults^20,22^ with MDD. Because human trial participation was restricted primarily to women, sex differences in human treatment response to creatine should be interpreted cautiously. Brain magnetic resonance spectroscopy studies, however, have shown that healthy women compared to healthy men have significantly lower concentrations of creatine phosphate, the phosphorylated form of creatine, which is involved in frontal lobe energy metabolism^58^. This phenomenon may be attributed, at least in part, to estrogen’s role in creatine homeostasis as a stimulant of creatine kinase activity^14,59^.

In contrast, it is possible that the interactive effects of estrogen and creatine on depression vary across a woman’s lifespan based on reproductive stage and hormone cycle phase. In addition to their effects on areas of the brain related to reproduction, ovarian steroids have widespread neurological impacts including on serotonin pathways, catecholamine neurons, the basal forebrain cholinergic system, and the hippocampal formation^60^. The onset of puberty marks a dramatic increase in the prevalence of depression in females compared to males, from approximately equal^61^ to twice that of males (prevalence ratio = 1.3–3.1; lifetime gender ratio = 2.1)^62^. In midlife, evidence has emerged that points to a temporal link between depression onset and the menopause transition in a subset of women, and the efficacy of exogenous estrogen administration during peri-menopause^63^. However, at present the existence of menopause-associated depression remains controversial, due to inconsistency in the methods used to characterize reproductive staging and evaluate psychiatric conditions in studies of midlife women with depression^64^. Importantly, estrogen^65^ and creatine^66^ both possess the ability to target mitochondrial function in the brain, raising the possibility they may interact to potentiate one another’s effects in treating or preventing depression. This relationship, however, is complicated by neuroimaging evidence demonstrating that a woman’s brain creatine concentration is a ‘state’ marker, which varies with systemic estrogen levels in the menstrual, follicular, and luteal phases^67^. Thus to elucidate the relationship between, and the respective contributions of estrogen and creatine to depression would require a measure of systemic estrogen and magnetic resonance spectroscopy brain scanning to quantitate in vivo brain creatine, with both performed on the day depression status is assessed. Future studies will be needed to explore how dietary creatine is related to mood symptoms across both the menstrual cycle and the lifespan, including pre-pubertal girls, adolescent females, women of childbearing age, as well as peri-menopausal and post-menopausal women.

The current study also provides preliminary evidence that the relationship between dietary creatine and depression risk may be moderated by age and use of antidepressant/anxiolytic medications. First, the strength of the association between dietary creatine and depression was strongest among participants in the youngest age category (20–39 years). While little is known about potential differences in the antidepressant effectiveness of creatine across the lifespan, one hypothesized mode of action for creatine’s antidepressant effects is through improvements to brain energy metabolism and capacity. Brain total creatine concentrations are reported to increase with age suggesting that the brain’s capacity for bioenergetics may also increase with age^68^. If correct, antidepressant effectiveness of creatine supplementation should be expected to differ across the lifespan. Second, an inverse relationship between dietary creatine intake and depression risk was identified in individuals not taking antidepressant or anxiolytic medications. While the implications of this finding are not clear, it may indicate that dietary creatine’s antidepressant effects are independent of antidepressant or anxiolytic medication use as has been reported in studies of female rats^24,25^.

This study’s findings may also have implications for adults who limit their consumption of animal protein, the principal source of creatine in the North American diet^69^. Previous epidemiologic research suggests that individuals consuming low animal protein diets may be at an increased risk of depression^70–73^. For example, a study using data from the German Health Interview and Examination Survey compared rates of depressive disorders among completely vegetarian, predominately vegetarian, and non-vegetarian participants and reported an increased prevalence of depressive disorders among completely vegetarians controlling for sociodemographic factors^72^. In contrast, other epidemiologic research identified no such link between a vegetarian diet and depression^74,75^.

Experimental treatment dosages of creatine used in pilot and clinical trial depression studies range from 2 to 10 g/day^17–22^. Such dosages are consistent with the daily creatine amounts recommended for use as a sports supplement during the maintenance phase (standard protocol for creatine use as an exercise supplement also includes an initial “loading phase” of 20 g of creatine daily for 5–7 days)^76^ and as therapeutic treatment for some muscle (e.g. inflammatory myopathies)^77^ and central nervous system disorders (e.g. Alzheimer disease, Parkinson disease, Huntington disease)^78^. In contrast, dietary creatine amounts consumed by study participants ranged from 0–3.16 g/day and averaged 0.54 g/day. Therefore, the current study’s findings suggest that even modest increases in creatine intake may have therapeutic value. For example, participants in the third quartile of dietary creatine intake consumed 5.12 ounces of animal protein on average, which approximates established US nutritional guidelines (i.e. 5.5 oz/day)^79^ and corresponds to an average daily dietary creatine intake of 0.57 g. Supplemental use of creatine at more modest levels is also supported by research suggesting the potential for creatine oversaturation as creatine intake exceeding a certain threshold may adversely influence other brain metabolite levels such as N-acetylcholine, choline, and ATP^80,81^. The optimal dosage of supplemental creatine for clinical use has yet to be established.

Study limitations include the assumption that dietary data collected by NHANES provides a valid estimate of participants’ dietary intake, an assumption that has been subject to challenge as self-reported dietary intake interviews have been reported to underreport total daily caloric intake^82^ and be susceptible to measurement error^83^. However, the 24 h, 2-day dietary recall technique employed by NHANES is often considered the preferred approach for measuring actual dietary intake in large population studies because it is minimally burdensome, minimizes risk of recall bias, does not require advanced literacy, and has been shown to produce valid estimates of average usual dietary intake at the population and sub-population levels^84^.

Average daily adult consumption of creatine in the U.S. diet is estimated to be around 1 g/day for men and 0.70 g/day for women^85^ while the corresponding estimates in the current study are 0.67 g and 0.42 g for adult men and women, respectively. Average total meat intake in the current study was estimated to be 138.9 g/person/day. This calculation is similar to another recently published NHANES-based estimate of 128 g of total meat/person/day^86^. Our literature review^36–45^ demonstrates that creatine contents vary widely within and among animal protein subgroups depending on the experiment (Supplementary Tables S1 and S2). Hence, the lower daily dietary creatine estimates from the current study compared to Brosnan et al^85^. may reflect differences in the underlying assumptions concerning the creatine content of animal protein sources. We also excluded non-meat protein sources of creatine, such as milk and cranberries, from dietary creatine calculations as their contribution to creatine intake in the US diet is relatively negligible (0.1 g/kg and 0.02 g/kg, respectively)^36^.

The prevalence of supplemental creatine use among adults in the current study (0.09%) is a magnitude lower than a comparable population-based estimate by Kaufmann et al^49^. Study discrepancies may be attributed to study design differences including different definitions of adulthood (18 + years vs. 20+ years), period effects (survey completed in 1998–1999 vs. 2005–2012), and data collection method (telephone interview vs. in-person assessment). While concerted efforts were made to control for potential confounders, additional lifestyle choices that may influence mood (i.e., additional dietary supplements, sleep habits) were beyond the scope of consideration for this study.

Creatine intake from naturally occurring dietary protein sources is invariably accompanied by additional nutritional components (i.e., Vitamin B12, tyrosine, tryptophan, and n-fatty acids) depending on the specific source of animal protein consumed. These nutrients may work independently from or additively/synergistically with creatine to reduce depression risk. Because dietary fish is a creatine source particularly high in n-fatty acids, we examined whether the association between depression risk and dietary creatine differed in models also adjusted for ounces of fish intake (total and fish high in n-fatty acids). The inclusion of ounces of fish consumed did not impact study findings however additional investigation is merited to explore potential interactive effects of creatine with other nutrients on depression risk.

The current study used cross-sectional data collected through NHANES and was designed to measure epidemiologic associations rather than causal relationships. While a stepwise, inverse relationship was identified between increasing dietary creatine consumption and depression risk, the presence of a causal relationship and the directionality of the association (i.e. MDD may reduce dietary intake and therefore our finding that reduced creatine consumption is associated with depression may be a consequence of MDD and not a precursor) cannot be assumed from this study design.

Study strengths include the use of data from NHANES, which is the only U.S.-based survey of its kind providing validated health, demographic, examination and dietary information on a nationally representative sample. In particular, NHANES (1) used the PHQ-9, a valid and reliable instrument to identify persons with MDD, (2) collected critical confounders, and (3) provided the ability to collapse data across multiple years, maximizing study size, power, and precision.

The association between diet and depression remains an active area of investigation; study findings support a relationship between dietary creatine and depression. By identifying an inverse, stepwise association between depression and dietary creatine consumption on a population level, study results support findings from creatine clinical trials and suggest that daily creatine intake, within the context of the average North American diet, may influence mood. Likewise, the increased propensity of female NHANES participants to benefit from dietary creatine intake reinforces prior animal studies demonstrating a sex bias in treatment response to creatine supplementation. Collectively, these results justify further exploration into creatine’s antidepressant-like mechanisms, particularly as they may relate to sex and age-specific effects.

## Supplementary information

Supplemental materials
