# Supplementary material for: Dietary creatine intake and depression risk among U.S. adults
Source: Transl Psychiatry. 2020 Feb 3;10:52. doi: 10.1038/s41398-020-0741-x (PMC7026167; doi:10.1038/s41398-020-0741-x)
Supplement: Supplementary file 1 — Supplemental materials [file 41398_2020_741_MOESM1_ESM.docx]

**Supplementary Tables**

S1. Calculation of average grams of creatine per kilograms of protein from meat, poultry, fish and processed meat sources based on published data.

S2. Calculation of average grams of creatine per ounce of MyPyramid Equivalents^19^ database animal protein subgroups.

S3. Sensitivity analysis investigating the association between two-day average dietary creatine intake and risk of depression among adult NHANES 2005-2012 participants using models adjusted for average ounces consumed of fish high in n-3 fatty acids (Model I) and total ounces of fish consumed (Model II)

| **S1. Calculation of average grams of creatine per kilograms of protein from meat, poultry, fish and processed meat sources based on published data.** | | | | | | | | | | | | | | | | | |
| --- | --- | --- | --- | --- | --- | --- | --- | --- | --- | --- | --- | --- | --- | --- | --- | --- | --- |
| Reference^1^ | Beef | Beef heart | Bacon | Chicken | Cod | Deli meats | Fish | Frank-furter | Herring | Lamb | Pork | Salmon | Sausage | Tuna | Turkey | Veal | Venison |
| 1 | 4.50 |  |  |  | 3.00 |  |  |  | 8.25 |  | 5.00 | 4.50 |  | 4.00 |  |  |  |
| 2 | 4.90 |  |  |  |  |  |  |  |  |  |  |  |  |  |  |  |  |
| 3 | 5.30 |  |  | 4.82 |  |  |  |  |  | 4.35 | 4.99 |  |  |  | 2.84 | 4.88 | 4.40 |
| 4 |  |  |  |  |  |  | 3.85 |  |  |  |  |  |  |  |  |  |  |
| 5 |  |  |  |  |  |  | 4.48 |  |  |  |  |  |  | 6.45 |  |  |  |
| 6 | 6.33 |  |  | 3.99 |  |  | 7.06 |  |  |  | 4.77 |  |  |  | 4.64 |  |  |
| 7 |  |  |  |  |  |  |  |  |  |  |  |  | 2.40 |  |  |  |  |
| 8 |  |  | 3.00 |  |  | 2.07 |  | 0.75 |  |  |  |  |  |  |  |  |  |
| 9 |  |  |  |  |  |  |  | 1.23 |  |  |  |  |  |  |  |  |  |
| 10 |  | 2.98 |  |  |  |  |  |  |  |  |  |  |  |  |  |  |  |
| Average | 4.85^2^ | 2.83^2^ | 3.00^3^ | 4.19^2^ | 2.85^2^ | 2.07^3^ | 4.87^2^ | 0.99^3^ | 7.84^2^ | 4.13^2^ | 4.69^2^ | 4.28^2^ | 2.28^2^ | 4.96^2^ | 3.55^2^ | 4.64^2^ | 4.18^2^ |
| ^1^See S1 references below for list of references | | | | | | | |  |  |  |  |  |  |  |  |  |  |
| ^2^Average decreased by 5% to account for cooking^11^ | | | | | | | |  |  |  |  |  |  |  |  |  |  |
| ^3^Average was not decreased by 5% because product was already cooked prior to testing | | | | | | | | | | | |  |  |  |  |  |  |

| **S2. Calculation of average grams of creatine per ounce of MyPyramid Equivalents^19^ database animal protein subgroups.** | | | | | | | |
| --- | --- | --- | --- | --- | --- | --- | --- |
| Individual meat item | M_MEAT^1^ | M_ORGAN^2^ | M_FRANK^3^ | M_POULT^4^ | M_FISH_HI^5^ | M_FISH_LO^6^ | M_MPF^7^ |
| Beef (g/kg) | 4.85 |  |  |  |  |  | 4.85 |
| Beef heart (g/kg) |  | 2.83 |  |  |  |  | 2.83 |
| Bacon (g/kg) |  |  | 3.00 |  |  |  | 3 |
| Chicken (g/kg) |  |  |  | 4.19 |  |  | 4.19 |
| Cod (g/kg) |  |  |  |  |  | 2.85 | 2.85 |
| Deli meats (g/kg) |  |  | 2.07 |  |  |  | 2.07 |
| Fish (general) (g/kg) |  |  |  |  | 4.87 |  | 4.87 |
| Frankfurter (g/kg) |  |  | 0.99 |  |  |  | 0.99 |
| Herring (g/kg) |  |  |  |  | 7.84 |  | 7.84 |
| Lamb (g/kg) | 4.13 |  |  |  |  |  | 4.13 |
| Pork (g/kg) | 4.69 |  |  |  |  |  | 4.69 |
| Salmon (g/kg) |  |  |  |  | 4.28 |  | 4.28 |
| Sausage (g/kg) |  |  | 2.28 |  |  |  | 2.28 |
| Tuna (g/kg) |  |  |  |  | 4.96 |  | 4.96 |
| Turkey (g/kg) |  |  |  | 3.55 |  |  | 3.55 |
| Veal (g/kg) | 4.64 |  |  |  |  |  | 4.64 |
| Venison (g/kg) | 4.18 |  |  |  |  |  | 4.18 |
| Average g/kg protein | 4.50 | 2.83 | 2.09 | 3.87 | 5.49 | 2.85 | 3.89 |
| Average g/oz protein | 0.13 | 0.08 | 0.06 | 0.11 | 0.16 | 0.08 | 0.11 |
| ^1^ounces of cooked lean meat from beef, pork, veal, lamb, and game | | | | |  |  |  |
| ^2^ounces of cooked lean meat from all types of organ meats, including that from beef, pork, veal, lamb, game, poultry, and fish | | | | | | | |
| ^3^ounces of cooked lean meat from frankfurters, sausages, and luncheon meats | | | | | |  |  |
| ^4^ounces of cooked lean mean from chicken, turkey, and other poultry | | | | |  |  |  |
| ^5^ounces of cooked lean meat from fish, shellfish, and other seafood that are high in the n-3 fatty acids eicosapentaenoic acid and docosahexaenoic acid | | | | | | | |
| ^6^ounces of cooked lean meat from fish, shellfish, and other seafood that are high in the n-3 fatty acids eicosapentaenoic acid and docosahexaenoic acid | | | | | | | |
| ^7^ounces of cooked lean meat from beef, pork, veal, lamb, game, organ meats, frankfurters, sausages, luncheon meat, poultry, fish, and shellfish | | | | | | | |

| S3.^1^ Sensitivity analysis investigating the association between two-day average dietary creatine intake and risk of depression among adult NHANES 2005-2012 participants using models adjusted for average ounces consumed of fish high in n-3 fatty acids (Model I) and total ounces of fish consumed (Model II) | | | | | | |
| --- | --- | --- | --- | --- | --- | --- |
|  |  | Model I-Adjusted model including average ounces consumed of fish high in n-3 fatty acids^2^ | |  | Model II-Adjusted model including total ounces of fish consumed^3^ | |
| Population |  | Odds Ratio (95% CI) | p |  | Odds Ratio (95% CI) | p |
| Entire population |  | 0.70 (0.54-0.92) | 0.01 |  | 0.70 (0.52-0.94) | 0.02 |
| Abbreviations: CI, confidence interval | | | | | | |
| ^1^Models include imputed data | | | | | | |
| ^2^Models adjusted for income to poverty ratio, race/ethnicity, sex, age, education level, BMI, healthcare access, smoking status, total moderate-to-vigorous minutes of physical activity per week, antidepressant/anxiolytic medication use, and two-day average fish high in n-3 fatty acid consumption | | | | | | |
| ^3^Models adjusted for income to poverty ratio, race/ethnicity, sex, age, education level, BMI, healthcare access, smoking status, total moderate-to-vigorous minutes of physical activity per week, antidepressant/anxiolytic medication use, and two-day average total fish consumption | | | | | | |

**S1 references**

1. Balsom PD, Söderlund K, Ekblom B. Creatine in humans with special reference to creatine supplementation. *Sports Med* 1994; **18**(4): 268–280.
2. Camara AA, Arn KD, Reimer A, Newburgh LH. The twenty-four hourly endogenous creatine clearance as a clinical measure of the functional state of the kidneys. *J Lab Clin Med* 1951; **37**: 743–763.
3. Gibis M, Weiss J. Impact of precursors creatine, creatinine, and glucose on the formation of heterocyclic aromatic amines in grilled patties of various animal species. *J Food Sci* 2015; **80**(11): C2430-C2439.
4. Hughes RB. Chemical studies on the herring (Clupea harengus). IV.—Creatine in herring flesh, and its behaviour during heat processing.  *J Sci Food Agric* 1960; **11**(12): 700–705.
5. Marsh NL, Iwaoka WT, Mower HF. Formation of mutagens during the frying of Hawaiian fish: correlation with creatine and creatinine content. *Mutat Res* 1990; **242**(3): 181–186.
6. Pais P, Salmon CP, Knize MG, Felton JS. Formation of mutagenic/carcinogenic heterocyclic amines in dry-heated model systems, meats, and meat drippings. *J Agric Food Chem* 1999; **47**(3): 1098–1108.
7. Krӧckel L, Jira W, Kϋhne D, Mϋller W. Creatine blooms on the surface of prepacked fermented sausages. *Eur Food Res Technol* 2003; **217**(1): 1–3.
8. Puangsombat K, Gadgil P, Houser TA, Hunt MC, Smith JS. Heterocyclic amine content in commercial ready to eat meat products. *Meat Sci* 2011; **88**(2): 227–233.
9. Del Campo G, Gallego B, Berregi I, Casado J. Creatinine, creatine and protein in cooked meat products. *Food Chemistry* 1998; **63**(2): 187–190.
10. Purchas R, Rutherfurd SM, Pearce PD, Vather R, Wilkinson BHP. Concentrations in beef and lamb of taurine, carnosine, coenzyme Q10, and creatine. *Meat Sci* 2004; **66**(3): 629–637.

11. Busch S.The amount of creatine in meat. 2015. Available at https://www.livestrong.com/article/524782-the-amount-of-creatine-in-meat/. Accessed on December 22, 2017.
